# An MRI-defined measure of cerebral lesion severity to assess therapeutic effects in multiple sclerosis

**DOI:** 10.1007/s00415-015-8009-8

**Published:** 2016-01-11

**Authors:** Gloria Kim, Shahamat Tauhid, Sheena L. Dupuy, Subhash Tummala, Fariha Khalid, Brian C. Healy, Rohit Bakshi

**Affiliations:** Department of Neurology, Brigham and Women’s Hospital, Laboratory for Neuroimaging Research, Partners MS Center, Harvard Medical School, Boston, MA USA; Department of Radiology, Brigham and Women’s Hospital, Laboratory for Neuroimaging Research, Partners MS Center, Harvard Medical School, Boston, MA USA; Laboratory for Neuroimaging Research, One Brookline Place, Brookline, MA 02445 USA

**Keywords:** MRI, Multiple sclerosis, Glatiramer acetate, Brain atrophy, Composite scale

## Abstract

Assess the sensitivity of the Magnetic Resonance Disease Severity Scale (MRDSS), based on cerebral lesions and atrophy, for treatment monitoring of glatiramer acetate (GA) in relapsing-remitting multiple sclerosis (MS). This retrospective non-randomized pilot study included patients who started daily GA [*n* = 23, age (median, range) 41 (26.2, 53.1) years, Expanded Disability Status Scale (EDSS) score 1.0 (0, 3.5)], or received no disease-modifying therapy (noDMT) [*n* = 21, age 44.8 (28.2, 55.4), EDSS 0 (0, 2.5)] for 2 years. MRDSS was the sum of* z*-scores (normalized to a reference sample) of T2 hyperintense lesion volume (T2LV), the ratio of T1 hypointense LV to T2LV (T1/T2), and brain parenchymal fraction (BPF) multiplied by negative 1. The two groups were compared by Wilcoxon rank sum tests; within group change was assessed by Wilcoxon signed rank tests. Glatiramer acetate subjects had less progression than noDMT on T1/T2 [(median* z*-score change (range), 0 (−1.07, 1.20) vs. 0.41 (−0.30, 2.51), *p* = 0.003)] and MRDSS [0.01 (−1.33, 1.28) vs. 0.46 (−1.57, 2.46), *p* = 0.01]; however, not on BPF [0.12 (−0.18, 0.58) vs. 0.10 (−1.47,0.50), *p* = 0.59] and T2LV [−0.03 (−0.90, 0.57) vs. 0.01 (−1.69, 0.34), *p* = 0.40]. While GA subjects worsened only on BPF [0.12 (−0.18, 0.58), *p* = 0.001], noDMT worsened on BPF [0.10 (−1.47, 0.50), *p* = 0.002], T1/T2 [0.41 (−0.30, 2.51), *p* = 0.0002], and MRDSS [0.46 (−1.57, 2.46), *p* = 0.0006]. These preliminary findings show the potential of two new cerebral MRI metrics to track MS therapeutic response. The T1/T2, an index of the destructive potential of lesions, may provide particular sensitivity to treatment effects.

## Introduction

MRI has provided a range of tools to define pathological changes in the brain and spinal cord in patients with multiple sclerosis (MS) [1]. Prior studies have combined cerebral MRI lesion and atrophy measures to create composite scales to increase sensitivity, provide a comprehensive assessment of disease status, and, in turn, provide more clinical relevance than individual MRI measures on their own [2–11]. The first such measure, the Z4 score, combined measures of disease activity and disease severity, and has been applied to assess treatment response [2, 4, 8]. These MS-MRI composite scales have shown higher effect sizes in their relationship to clinical status or longitudinal change as compared to established individual MS-MRI measures [5–7, 11, 12]. Building on this previous work, we tested a cerebral MRI composite scale focusing on MS disease severity as shown by lesions and atrophy which also includes an assessment of the destructive potential of individual lesions, the intrasubject ratio of T1 hypointense to T2 hyperintense lesion volume (T1/T2). Known as the Magnetic Resonance Disease Severity Scale (MRDSS) [5], this tool has shown higher effect sizes in differentiating relapsing-remitting (RR) from secondary progressive (SP) patients and higher longitudinal sensitivity than the component MRI measures on their own [5, 7]. To date, the role of the MRDSS in treatment monitoring has not been tested.

Glatiramer acetate (GA) is an established disease-modifying therapy (DMT) for RRMS [13–16]. GA is known to effectively reduce relapse rates and the appearance of new gadolinium (Gd)-enhancing and T2 hyperintense lesions [16]. In addition, GA limits the progression of destructive aspects of the disease including the rate of brain atrophy [17] and the evolution of new Gd-enhancing lesions converting to chronic T1 hypointensities (“black holes”) [18]. The goal of the present pilot study was to evaluate the 2-year longitudinal sensitivity of the MRDSS in comparing GA-treated to untreated patients with RRMS. Furthermore, this study provided the unique opportunity to assess the role of T1/T2 in tracking therapeutic response.

## Methods

### Subjects

Baseline demographic and clinical characteristics are summarized in Table 1. This was a retrospective non-randomized two-arm observational exploratory study. Given the sample size and study design, the results should be considered preliminary. All subjects were identified by chart review using the following inclusion criteria: RRMS [19], age 18–55, and an Expanded Disability Status Scale (EDSS) [20] score 0–5. Among the 44 patients with RRMS, two groups were identified based on DMT use during the 2-year observation period: (1) GA-treated (*n* = 23) and (2) no DMT (noDMT, *n* = 21), i.e. patients who remained off DMT. Patients were required to have a baseline and 2-year brain MRI available. Clinical evaluation, including EDSS scoring [20] and timed 25 foot walk (T25FW) [21], were assessed by the treating neurologist at the Partners MS Center. When comparing groups on baseline clinical and demographic characteristics, the noDMT group showed a trend to a higher percentage of women (*p* = 0.07) and higher age (*p* = 0.09), and a significantly higher disease duration (*p* = 0.008) and lower EDSS score (*p* = 0.048). This study was approved by our institution’s research ethics committee and was performed in accordance with the ethical standards laid down in the 1964 Declaration of Helsinki and its later amendments.

**Table 1:** Subjects’ characteristics and clinical findings at baseline and 2-year follow-up

	Glatiramer acetate	NoDMT	*p* value
Baseline			
Number of patients	*n* = 23	*n* = 21	–
Women, number (%)	15 (65)	19 (90)	0.07
Age (years)	41.3 (26.2, 53.1)	44.8 (28.2, 55.4)	0.09
Disease duration^a^ (years)	1.8 (0.3, 20.3)	6.5 (0.4, 33.5)	0.008
EDSS score	1.0 (0, 3.5)	0 (0, 2.5)	0.048
T25FW (s)	4.2 (3.0, 5.6)	5.0 (3.4, 6.0)	0.32
Follow-up			
EDSS score	1.0 (0, 3.0)	0 (0, 2.0)	0.39
T25FW (s)	4.2 (2.7, 6.0)	4.5 (3.5, 5.7)	0.07
On-study annualized relapse rate (mean)	0.13	0.12	0.88

Key: values are median (range), unless otherwise indicated

The *p* value for women—Fisher’s exact test, for on-study relapses—Poisson regression, and the other *p* values—Wilcoxon rank sum tests

*NoDMT* not receiving disease-modifying therapy, *RRMS* relapsing-remitting multiple sclerosis, *EDSS* Expanded Disability Status Scale, *T25FW* timed 25 foot walk

^a^Time since first symptoms

### MRI acquisition

All patients underwent baseline and follow-up 1.5 T brain MRI on a fleet of Signa scanners (General Electric, Milwaukee, WI) at our institution. All scans covered the whole brain in the axial plane and included T1-weighted spin-echo [repetition time and echo time (TR/TE): 550–783/20 ms] and T2-weighted conventional spin-echo dual-echo series (TR/TE1/TE2: 2750–3000/30/80 ms), with voxel sizes of either 0.9375 × 0.9375 × 3 mm or 0.8594 × 0.8594 × 3 mm, and no inter-slice gaps. The T1-weighted series was repeated 5–7 min after the intravenous infusion of single-dose Gd. MRI analysis was performed by observers who were unaware of the clinical details.

### MRI analysis

#### T2 hyperintense lesion volume and whole brain atrophy

Using the dual-echo T2-weighted images, the T2 hyperintense lesion volume (T2LV) and the normalized whole brain volume (brain parenchymal fraction—BPF) were calculated by an automated template-driven segmentation (TDS+) [22].

#### T1 hypointense lesion volume/T2 hyperintense lesion volume ratio

T1 hypointense lesions were initially identified and traced by a trained observer using a semi-automated edge-finding tool in Jim software (v.7; Xinapse Systems, West Bergholt, UK; http://www.xinapse.com). Each lesion and its contour was then confirmed by an experienced observer. T1 hypointense lesions were defined as appearing hypointense to the surrounding white matter, with corresponding hyperintensity on both of the dual-echo images. The lesions were also required to show non-enhancement on post-Gd images. To assess the destructive potential of lesions, the ratio of the T1 hypointense lesion volume to the T2LV (T1/T2) was calculated for each patient. This was used in favor of the total T1 hypointense lesion volume (T1LV) based on our previous work showing high co-linearity between T1LV and T2LV [5].

### Calculation of MRDSS

Magnetic Resonance Disease Severity Scale was derived by calculating *z*-scores for appropriate transformations of each component as in the original paper [5], and the mean and standard deviation used in the *z*-score calculation were the values from the original paper to ensure that these were representative of a typical clinic-based MS population [5]. For the BPF, no transformation was required, but the *z*-score was multiplied by −1 so that higher values of −zBPF represented lower BPF and worse disease severity. For T2LV, a log transformation was used, and the *z*-score was referred to as zT2LV. Finally, for the T1/T2, the logistic transformation was used, and the *z*-score was named zT1/T2. Subjects with T1/T2 of 0 were assigned a value more extreme (zT1/T2 = −2.5) than the smallest observed value (zT1/T2 = −1.96). We note that all values for T1/T2 and T2LV were rounded to two decimal places. To combine the *z*-scores, the following equation was used:

$$ {\text{zMRDSS}} = - {\text{zBPF}} + {\text{zT2LV}} + {\text{zT1}}/{\text{T2}} $$

The zMRDSS was not rescaled to lie between 0 and 10 as in the original paper because observations in the present sample would have been outside of this range. Rather, the zMRDSS score was used for analysis. For simplicity purposes, the zMRDSS is referred to often as MRDSS throughout the remaining sections of the paper. This version of the MRDSS has been referred to as “MRDSS1” in a subsequent paper [10].

### Statistical analysis

In order to compare the GA treated subjects to the untreated subjects in terms of MRI measures at baseline, year 2 and on-study changes in MRI measures, Wilcoxon rank sum tests were performed. To determine if there were within group changes in MRI measures over time, Wilcoxon signed rank tests were used. For clinical outcomes, the EDSS and T25FW in each group were compared at baseline and year 2 using a Wilcoxon rank sum test. To compare the groups on changes over time in clinical outcomes, a mixed effects ordinal logistic regression model was used. Finally, to compare the number of on-study relapses, a Poisson regression model was used. A *p* < 0.05 was considered significant; a *p* > 0.05 but <0.10 was considered a trend to significance. All statistical analysis was completed in the statistical package R (http://www.r-project.org) or Stata (version 14).

## Results

### Baseline and follow-up MRI differences in the GA and noDMT groups

When evaluating the MRI differences between the GA and noDMT groups at baseline and follow-up, no significant differences were found (Table 2). Only the T1/T2 difference at follow-up showed a trend towards significance (lower in the GA group).

**Table 2:** MRI findings at baseline and 2-year follow-up

	Glatiramer acetate	NoDMT	*p* value
Baseline			
BPF	0.899 (0.808, 0.934)	0.878 (0.761, 0.950)	0.43
T2LV (ml)	2.57 (0.75, 9.31)	3.51 (1.00, 28.69)	0.29
T1/T2	0.24 (0.01, 0.91)	0.20 (0, 0.79)	1
−zBPF	−1.24 (−1.88, 0.47)	−0.84 (−2.18, 1.36)	0.43
zT2LV	−0.79 (−2.26, 0.74)	−0.42 (−1.91, 2.07)	0.29
zT1/T2	0.59 (−1.92, 3.11)	0.42 (−2.50, 2.39)	1
zMRDSS	−1.55 (−4.35, 3.38)	−0.78 (−4.15, 4.28)	0.46
Follow-up			
BPF	0.888 (0.784, 0.936)	0.873 (0.754, 0.941)	0.54
T2LV (ml)	2.52 (0.62, 9.34)	2.83 (0.84, 26.67)	0.35
T1/T2	0.23 (0.02, 0.7)	0.33 (0.02, 0.98)	0.07
−zBPF	−1.03 (−1.92, 0.93)	−0.74 (−2.01, 1.49)	0.54
zT2LV	−0.82 (−2.48, 0.74)	−0.68 (−2.12, 1.99)	0.35
zT1/T2	0.55 (−1.41, 2.04)	0.91 (−1.41, 4.26)	0.07
zMRDSS	−1.15 (−3.75, 2.62)	−0.55 (−3.53, 5.64)	0.16

Key: median (range)

All *p* values are from Wilcoxon rank sum tests comparing the groups. The negative zBPF is shown so that the direction matched the others (a positive score indicates advancing disease). The MRDSS was calculated as a zMRDSS because the original scaling of the MRDSS to a 0–10 scale led to a MRDSS of >10 for a follow-up score

*NoDMT* not receiving disease-modifying therapy, *BPF* brain parenchymal fraction, *T2LV* total cerebral T2 hyperintense lesion volume, *T1/T2* ratio of T1 hypointense to T2 hyperintense lesion volume, *MRDSS* magnetic resonance disease severity scale, *z* standardized

### On-study changes in MRI measures in the GA and noDMT groups

**Fig. 1:**
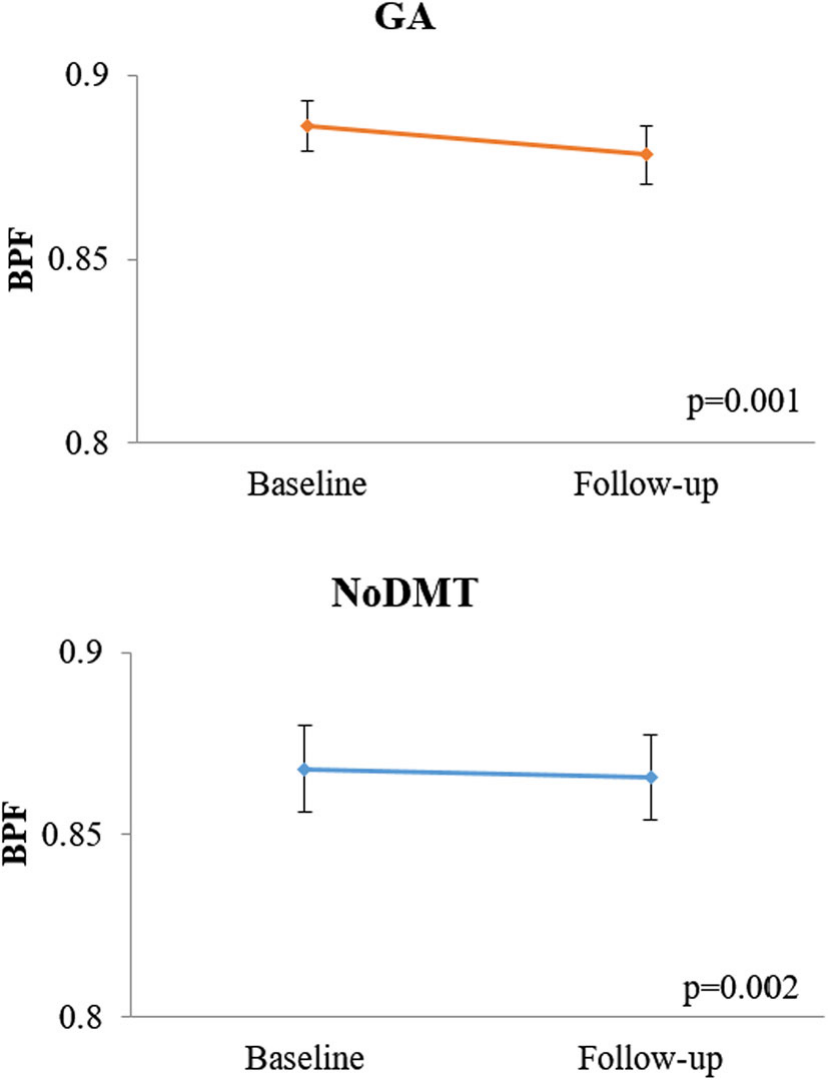
On-study brain atrophy in glatiramer acetate vs. untreated patients. Mean (±standard error of the mean) of brain parenchymal fraction (BPF) at baseline and 2-year follow-up. A lower score indicates advancing disease. Both glatiramer acetate (GA) and no disease modifying therapy (noDMT) cohorts showed significant decreases in BPF (i.e. no brain atrophy) from baseline to follow-up. The *p* values in the figure are from Wilcoxon signed rank tests for the within group change over time. Furthermore, when comparing the change in zBPF between the two groups, no difference was found (Wilcoxon rank sum test, *p* = 0.59)

**Fig. 2:**
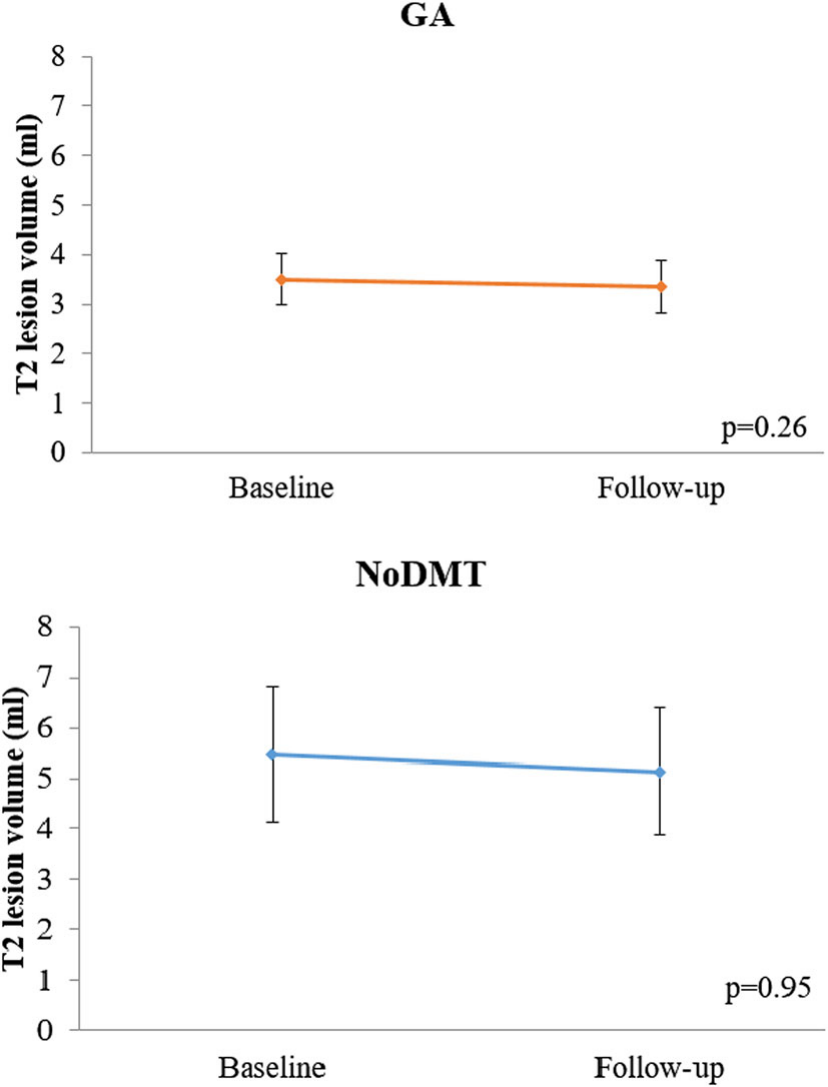
On-study T2 hyperintense lesion volume changes in glatiramer acetate vs. untreated patients. Mean (±standard error of the mean) of total cerebral T2 hyperintense lesion volume (T2LV) at baseline and 2-year follow-up. A higher score indicates advancing disease. Both glatiramer acetate (GA) and no disease modifying therapy (noDMT) cohorts showed no significant decreases from baseline to follow-up. The *p* values in the figure are from Wilcoxon signed rank tests for the within group change over time. Furthermore, when comparing the change in zT2LV between the two groups, no difference was found (Wilcoxon rank sum test, *p* = 0.40)

**Fig. 3:**
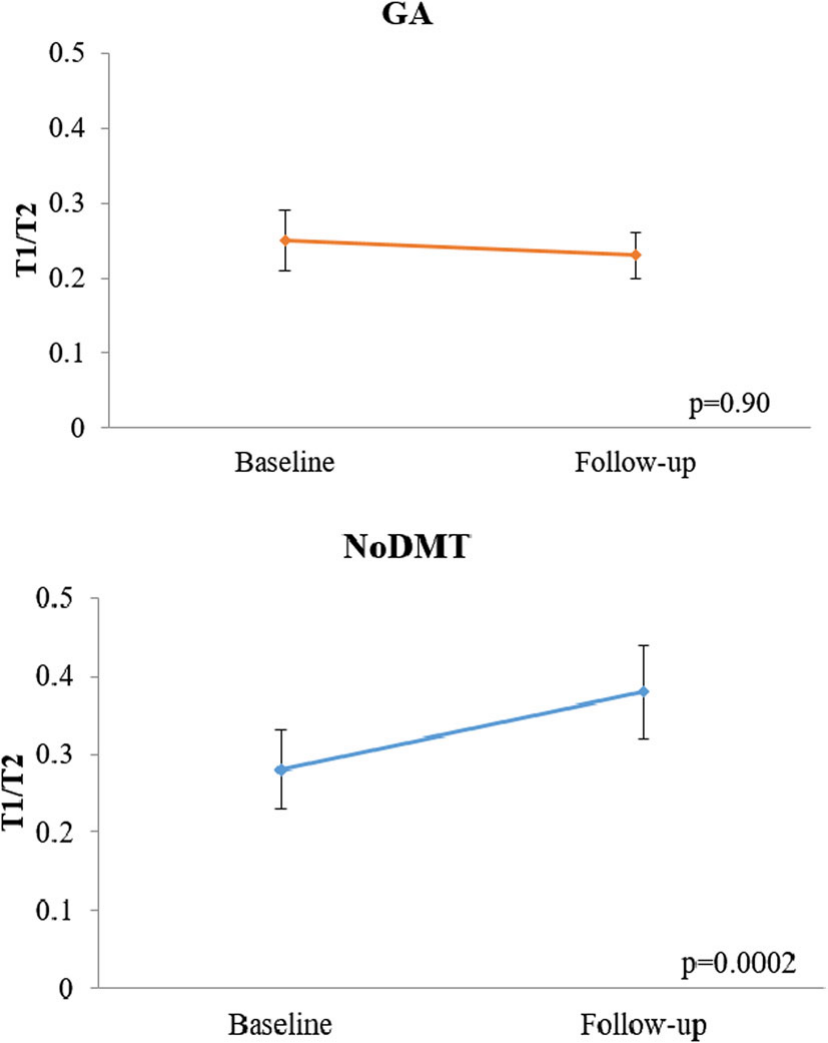
On-study T1/T2 ratio changes in glatiramer acetate vs. untreated patients. Mean (±standard error of the mean) of the ratio of total cerebral T1 hypointense to T2 hyperintense lesion volume (T1/T2) at baseline and 2-year follow-up. A higher score indicates advancing disease. The no disease modifying therapy (noDMT) group showed significant worsening, but the glatiramer acetate (GA) treated group did not. The *p* values in the figure are from Wilcoxon signed rank tests for the within group change over time. Furthermore, when comparing the change in zT1/T2 between groups, a difference was found favoring GA treatment (Wilcoxon rank sum test, *p* = 0.003)

**Fig. 4:**
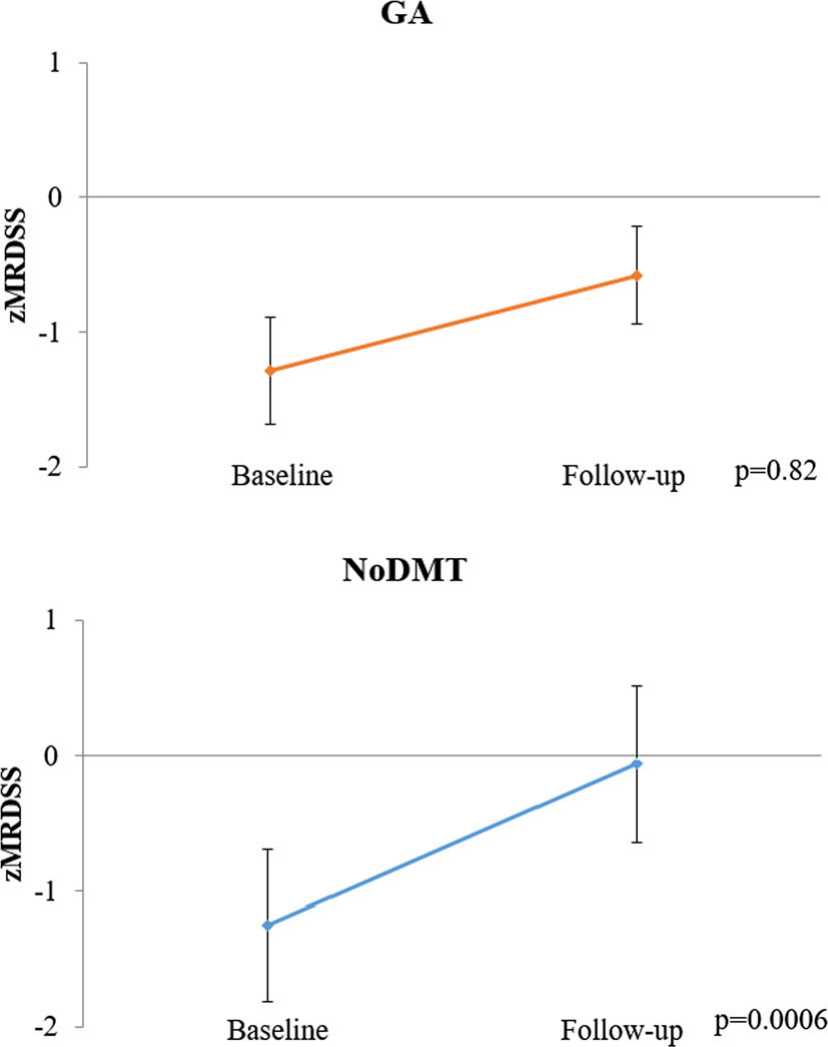
On-study MRDSS changes in glatiramer acetate vs. untreated patients. Mean (±standard error of the mean) of the magnetic resonance disease severity scale (zMRDSS) at baseline and 2-year follow-up. A higher score indicates advancing disease. The no disease modifying therapy (noDMT) group showed significant worsening, but the glatiramer acetate (GA) treated group did not. The *p* values in the figure are from Wilcoxon signed rank tests for the within group change over time. Furthermore, when comparing the change in zMRDSS between groups, a difference was found favoring GA treatment (Wilcoxon rank sum test, *p* = 0.01)

When comparing the 2-year on-study changes in MRI measures between the GA and noDMT groups (Table 3; Figs. 1, 2, 3, 4), subjects in the GA group had significantly less worsening of disease over the 2-year follow-up in terms of T1/T2 (Fig. 3) and MRDSS (Fig. 4). Considering the on-study changes within the GA cohort, a significant worsening was only seen in BPF [0.12 (−0.18, 0.58), *p* = 0.001). However, when assessing the on-study changes within the noDMT group, statistically significant worsening was seen in BPF [0.01 (−1.47, 0.50), *p* = 0.002], T1/T2 [0.41 (−0.30, 2.51), *p* = 0.0002], and MRDSS [0.46 (−1.57, 2.46), *p* = 0.0006]. Because the *p* value for the group comparison for the difference was more statistically significant for the T1/T2 than the other individual MRI components, this implies that the difference between treatment groups in MRDSS was driven by the T1/T2 change. This was confirmed by the observations in Table 3 that there was only a limited group difference in the change for the BPF or T2LV.

**Table 3:** MRI 2-year on-study changes

	Glatiramer acetate	*p* value	NoDMT	*p* value
−zBPF	0.12 (−0.18, 0.58)	0.001	0.10 (−1.47, 0.50)	0.002
zT2LV	−0.03 (−0.90, 0.57)	0.26	0.01 (−1.69, 0.34)	0.95
zT1/T2	0 (−1.07, 1.20)	0.90	0.41 (−0.30, 2.51)	0.0002
zMRDSS	0.01 (−1.33, 1.28)	0.82	0.46 (−1.57, 2.46)	0.0006

Key: median (range)

All *p* values are from Wilcoxon signed rank tests assessing whether there was a significant change over 2 years for each measure within each group. The negative zBPF is shown so that the direction matched the others (a positive score indicates advancing disease). The MRDSS was calculated as a zMRDSS because the original scaling of the MRDSS to a 0–10 scale led to a MRDSS of >10 for a follow-up score

*NoDMT* not receiving disease-modifying therapy, *BPF* brain parenchymal fraction, *T2LV* total cerebral T2 hyperintense lesion volume, *T1/T2* ratio of T1 hypointense to T2 hyperintense lesion volume, *MRDSS* magnetic resonance disease severity scale, *z* standardized

### On-study clinical changes/relapses

On-study and follow-up clinical changes/relapse data are shown in Table 1. When comparing the groups in terms of on-study EDSS and T25FW change, a mixed effects ordinal regression model determined that there was no significant group difference in terms of change over time (*p* = 0.29 and *p* = 0.35, respectively). At follow-up, there was no difference between groups in EDSS score (*p* = 0.39). At follow-up, the T25FW trended to being lower in the GA vs. the noDMT group (*p* = 0.07). Patients in the GA group had an average of 0.26 clinical relapses over the 2 years (mean annualized attack rate = 0.13). This was 0.24 (mean annualized attack rate = 0.12) in the noDMT group. A Poisson regression model determined that there was no significant difference between the groups in terms of on-study relapses (*p* = 0.88).

## Discussion

In this pilot study, we explored the use of a cerebral MRI-based composite scale of disease severity, based on lesions and atrophy, the MRDSS [5, 7], to assess the response to GA in RRMS over 2 years. The main findings in this study are that the two established measures of lesions and atrophy, T2LV and BPF, did not show a difference between GA and an untreated group. However, the MRDSS was sensitive to a group difference, which was driven by the scale’s inclusion of a unique metric of the destructive potential of lesions, the intrasubject T1/T2 ratio. These preliminary results underscore the potential limitations of conventional MRI metrics for longitudinal monitoring and show the potential utility of a more comprehensive consideration of structural changes that may relate to disease evolution, particularly the representation of lesion destructive potential.

While the present study is the first to consider the use of MRDSS or T1/T2 to monitor treatment response in MS, the Z4 composite score has been tested in the past. In a clinical trial of roquinimex (linomide), the Z4 significantly distinguished the treatment groups at 3 months, and showed a similar pattern between the groups at 6 months. In a study evaluating the long term impact of GA therapy in a placebo-controlled cross-over trial, the Z4 discriminated patients well according to duration of therapy and clinical outcome [3]. In a placebo-controlled trial of anti-chlamydial therapy in RRMS, the Z4 increased in the placebo group and decreased in the antibiotic treatment group over 1 year [4]. These differences suggested a trend towards the benefit of therapy and were driven by the stabilization in the brain atrophy component of the scale. Unlike the Z4, our MRDSS uses a novel measure of lesion severity, the T1/T2 ratio, combined with overall burden of disease (T2LV) and brain atrophy (BPF).

Prior work in developing the MRDSS has shown several attractive features setting the stage for testing its sensitivity in treatment monitoring [5, 7]. First, the MRDSS showed a higher effect size in differentiating RR vs. SP MS phenotype groups when compared to the individual component MRI measures [5]. Second, the MRDSS showed a higher correlation with physical disability than what was seen with conventional MRI lesion load [5]. Third, in a 3-year longitudinal study, the MRDSS was more sensitive to change in both RR and SP phenotype groups than the individual component MRI measures [7]. Fourth, the MRDSS showed the largest effect size in differentiating cognitively preserved vs. cognitively impaired patients with MS when compared to the individual component MRI measures [10]. Finally, the standardization of measures that is necessary to derive the MRDSS most likely improves the clinical relevance [5]. The current results extend our previous observations in that the MRDSS detected a difference between treatment groups that was not apparent when considering the established measures of conventional lesion load or brain atrophy on their own. Taken together, these exploratory findings underscore the potential advantages of a comprehensive scale of MRI-defined disease severity that considers several related but different aspects of disease pathophysiology.

The MRDSS detected a group difference that was not apparent by considering changes in either T2LV or BPF. It was clear from examination of the data, that the group difference in MRDSS, suggesting a treatment effect of GA, was dominated by changes in the T1/T2. This is the intrasubject ratio of the volume of cerebral T1 hypointense to T2 hyperintense lesions. This ratio roughly equates to an index of the destructive potential of a patient’s lesions in that it is well known that chronic T1 hypointensity indicates severe destructive pathology (severe irreversible demyelination and axonal loss) [23–25]. Given that the scale is derived from a single time point scan, the chronicity of T1 hypointensity is not assured. But, the exclusion of Gd-enhancing lesions from our definition of T1 hypointense lesions reduced the likelihood of including transient/benign T1 hypointense lesions [26]. Furthermore, the high clinical relevance of the T1/T2 has been suggested by several previous studies. One study showed a higher T1/T2 in SP vs. RR MS, with higher effect sizes than T2LV or BPF differences [5]. In a 3-year longitudinal study, the T1/T2 was more sensitive to change in both RR and SP phenotype groups than T2LV or BPF [7]. In addition, the T1/T2 showed a larger effect size in differentiating cognitively preserved vs. cognitively impaired patients with MS when compared to T2LV [10]. The T1/T2 shows only moderate correlations with either T2LV or BPF [5], indicating its ability to detect divergent aspects of disease severity. The pathobiologic factors contributing to the tendency towards more destructive lesions in MS patients are unknown. One line of investigation relates to genetic predisposition [27, 28]. There is a relatively low co-linearity between T1/T2 and either T1LV, T2LV, or BPF [5]. Taken together, these observations suggest the unique role of the information provided by T1/T2 that may complement the evaluation of cerebral disease severity obtained by standard evaluations of lesion load and atrophy.

There are several lines of evidence regarding the effect of GA therapy in MS that may help to explain the selective effect on T1 hypointense-associated lesion characteristics suggested in this pilot study. While most of the available DMTs for MS act by reducing lymphocyte entry into the CNS, GA is thought to have a unique mechanism of action [29, 30]. Animal model and clinical studies show the effect of GA on shifting pro-inflammatory to anti-inflammatory immune actions [29, 30]. In addition to immunomodulatory effects, the drug may also promote the secretion of neurotrophins to enhance repair processes and remyelination [29]. Consistent with this hypothesis, a placebo-controlled phase III clinical trial showed that the percentage of new cerebral lesions evolving into chronic T1 hypointensities was lower in GA-treated vs. placebo-treated RRMS patients [18]. Such a treatment effect of GA has been confirmed in subsequent studies of different patient populations [31, 32]. In the present study, when comparing the change in zT1/T2 between groups, a significant difference was found favoring GA treatment vs. no treatment. This was the result of a significant increase in the T1/T2 in untreated patients but no change in GA patients over 2 years. Taken together, while preliminary and requiring confirmation in larger studies, these results suggest that GA can limit tissue destruction in lesions once they have formed leading to a reduced level of long term tissue injury.

In the present study, the GA and untreated groups did not differ on their rates of changes in T2LV or BPF over the 2-year observation period. This is in contrast to the results of large phase III placebo-controlled studies, in which GA reduced the rate of progression of T2LV in RRMS [14] or clinically-isolated demyelinating syndromes [33]. Our study, given the much smaller sample size and retrospective study design, may have been under-powered to show such effects. Regarding the effect of GA on limiting brain atrophy, results have been inconsistent [34], showing either a partial delayed effect [17, 35] or no effect [33, 36] in the above-referenced phase III studies. Furthermore, the sensitivity of brain atrophy as a longitudinal monitoring tool in the evaluation of MS therapies has been hampered by several factors [37, 38]. The limitations include the delayed effect of newly started DMT on atrophy (usually requiring a lag time of several months to a year), the partial effect (a maximum benefit of up to 40–50 % per year in the rate of reduction), and the confounding effects on brain volume of acute DMT-related or corticosteroid brain volume change (i.e. pseudoatrophy due to anti-inflammatory effects and fluid shifts) [37, 38]. Furthermore, other unexpected factors may alter brain volume measurements such as diurnal fluctuations [39]. Taken together, the above observations suggest it is perhaps not surprising that our study failed to detect any difference in T2LV or BPF change between the two groups in this small study.

Our study was not without several limitations. This work was exploratory and the findings should be considered preliminary. The “real world” subject groups may have been biased due to the retrospective study design and non-randomized treatment assignment. The groups were not ideally matched at study entry on male/female ratio, age, disease duration, and disability. This may have affected the results in that, for example, the higher disability, higher percentage of men, and lower disease duration at entry in the GA group may have led to a bias. Future studies should be properly designed to provide more definitive results which would allow extension and confirmation of our observations. Post-hoc analyses of phase III trial data would be particularly helpful to overcome several of these limitations. Advanced MRI techniques may prove more specific for use at a single time point in the identification of the most destructive lesions rather than relying on the volume of hypointensity on T1-weighted images [26, 40]. Finally, to better understand the full breadth of any potential treatment effects of GA, it would be of interest to incorporate MRI measures of gray matter and spinal cord pathology. Both of these aspects of disease severity have shown a benefit in improving the validity of MS-MRI composite scales [10, 11].
